# Impact of a Practical, Hands-On, Continuing Professional Development Course About AI in Health Care Professions Education on the Perceptions and Behaviors of Health Care Educators: Qualitative Case Study

**DOI:** 10.2196/87381

**Published:** 2026-06-23

**Authors:** Justin G Peacock, Jerusalem Merkebu, Anita Samuel

**Affiliations:** 1 Department of Radiology and Bioengineering, School of Medicine, Uniformed Services University of the Health Sciences, 4301 Jones Bridge Road, Bethesda, MD, 20814, United States, 1 (301) 295-3628; 2 Department of Health Professions Education, School of Medicine, Uniformed Services University of the Health Sciences, Bethesda, MD, United States

**Keywords:** artificial intelligence, AI, technology education, connectivism learning theory, experiential learning theory, health professions education, HPE, AI education, continuing professional development, CPD

## Abstract

**Background:**

Artificial intelligence (AI) is increasingly being used in many aspects of society, including health care and education. AI has the potential to enhance health care delivery, education, and administration. Health care trainees will be required to master these AI technologies. To teach trainees to effectively and ethically leverage AI technologies, educators must be appropriately trained and empowered to use these technologies.

**Objective:**

We developed a health professions education course to enable health care professionals to overcome their fears and concerns about integrating multimodal generative AI technologies in daily practice. The course was also designed to foster generative AI skills and confidence in educational, administrative, research, and clinical activities.

**Methods:**

Using a multimethod approach, we analyzed data gathered from three different sources using the 6-phase reflexive thematic analysis by Braun and Clarke. This involved familiarization with the data sources, generating initial codes, developing, refining, and defining the themes, and finally, writing up the results.

**Results:**

Our findings indicate that 16 of 21 (76%) learners initially described apprehension or fear toward AI technologies. After experiential engagement with AI technologies together with their peers, they were able to shift their perspectives and gain confidence to integrate AI tools in their daily practice.

**Conclusions:**

A brief 6-week continuing professional development course on the use of AI technologies for health care professional educators, focused on experiential and peer-based learning, resulted in shifts in perception, affect, and behavior toward AI technologies. It also propelled learners to shift increasingly outward in their discussion, application, and preliminary advocacy for AI technologies in their daily practice.

## Introduction

### Background

Artificial intelligence (AI) is transforming the way that health care is taught and practiced [1-9]. AI tools are increasingly being used for administrative, clinical, research, and educational purposes in health care. This broad adoption of AI technologies necessitates that health professionals learn about the basic principles of AI and the ethical application of AI in clinical and educational practice [1,2,10-15].

Health professions education (HPE) research on AI education is relatively new, with significant growth after 2018, particularly in the post-2022 ChatGPT (OpenAI) era [1,2]. Much of the educational research describing AI in HPE focuses on how different AI tools can be used in health care education for educators and learners, and a few studies exploring AI curricula development [1-8,16]. The described AI curricula in HPE generally focus on basic AI principles, including AI ethics, but few describe hands-on, practical, or peer-based approaches to incorporating AI technologies in HPE practice [5,6,16,17]. The reported courses or curricula often reported Likert score summative impacts, with limited qualitative response assessments [5,6,17]. Only one study, published after submission of this manuscript, reported on qualitative reflections three months after the course [17]. All the evaluations were level 1 to 2 on the Kirkpatrick Framework scale [18], impacting individual knowledge and skill, but only one reported limited data on post-course attitude and behavior changes (Kirkpatrick level 3), and none reported on wider organizational changes or patient outcomes (Kirkpatrick level 4) [2,5,6,16,17].

Along with the lack of research on AI in HPE, there is also a dearth of literature on the best ways to teach AI concepts to HPE professionals, the qualitative impact of those techniques on the learner’s experience, and how teaching methods may lead to higher-level Kirkpatrick curricular outcomes. Laupichler et al [4] note that “it is important to find out how different populations respond to different teaching opportunities and what teaching materials are appropriate for teaching AI content.” There is a critical need to educate HPE professionals on the ethical and effective use of AI, so that they can, in turn, educate health care trainees [1,7,10,11]. Without this training, educators will not be able to answer questions, address concerns, or model ethical behavior regarding the use of AI technologies.

Before discussing the theoretical framework for this manuscript, it is important to first explain what is meant by AI. AI, in its broadest terms, is the ability for machines and computer algorithms to perform functions typically associated with humans, such as prediction, comprehension, writing, or art [19]. This can range from current, probabilistic AI tools (narrow AI capable of specific tasks) to theoretical, future, autonomous AI technologies (general or broad AI capable of autonomous human intelligence). The range and scope of AI technologies is rapidly becoming incomprehensible and frightening for many health care professionals, with many wondering whether they will be replaced by algorithms [20].

This wide-ranging, all-encompassing, human-like, and threatening nature of AI technologies necessitates a new way of teaching that is different from other forms of technology education. Learners need to understand the basics of how these tools are developed, where these tools fail, the social and ethical implications of AI tool implementation, and, importantly, how they view their personal and professional role in the context of an AI-enabled future [8,20,21].

Given the unique nature of AI technologies, we propose that experiential learning theory principles may be more effective in teaching HPE learners about AI concepts [22-24]. Experiential learning theory, as proposed by David Kolb, characterizes learning as a holistic process in which knowledge is continuously created through the transformation of experience. This framework posits that learners do not merely absorb information, but actively construct understanding by progressing through a four-stage cycle. Applying experiential learning theory to HPE AI education, the four steps of concrete experience, reflective observation, abstract conceptualization, and active experimentation align well with effective AI learning methods [22].

As HPE learners work with the AI tools to develop a product (eg, quiz questions, images, and educational presentations), reflect on what they observed (eg, failure modes, benefits, and ease of use), think about their observations (eg, image tools struggle with text and more precise prompting leads to better outcomes), and then experiment on those ideas (eg, revise the prompt, incorporate meta-prompting to improve outcomes, and develop custom AI tools), then they begin a cyclic or spiral learning pattern, described by Kolb, that may lead to increased confidence and competence [22]. By recursively navigating this spiral, learners move toward higher levels of cognitive complexity, fostering the increased confidence and perceived competence necessary for professional mastery. We used an experiential learning theory lens to design the AI course and guide our primary research question.

### Research Gap, Aim, and Question

In summary, the disruptive nature of AI technologies in society has caused many HPE professionals to feel anxious and fearful about the unique, existential threat posed by AI technologies [20,25]. The HPE literature is limited in describing how to effectively teach HPE professionals about AI concepts in a way that leads to affective, behavioral, and organizational changes in their personal practice (eg, higher order Kirkpatrick levels). Additionally, there is significantly limited qualitative data about the impact of continuing professional development (CPD) AI curricula on personal and professional changes that may occur.

In this qualitative case study on a CPD course for HPE professionals on AI technologies, we aim to understand how elements of experiential learning theory applied to the teaching of AI concepts may lead learners to shifts in their perception, affect, and behaviors about AI technologies.

We will explore the following research question: how does a short, experiential CPD course on AI tools in HPE influence learners’ perceptions, affects, and behaviors toward AI technologies in their daily practice?

## Methods

### Ethical Considerations

This qualitative case study was deemed exempt by the Uniformed Services University (USU) institutional review board (IRB; #24‐19215).

Deidentified artifacts from the course, including the precourse survey instrument and course assignments, were considered exempt educational artifacts.

For the semistructured interviews, the interviewer obtained informed consent from participants before the interview and maintained participant privacy as described in the interview methodology section. Participants received no compensation.

### Study Design

This qualitative case study was conducted in the HPE program at USU. The HPE program offers a 6-week blended online course (HPE 912) that is offered primarily asynchronously, titled “AI in Health Professions Education: Implications for Educators and Leaders.” Study participants were students enrolled during the spring and summer semesters of 2024. The course objectives, schedule, and five written assignments are attached (Multimedia Appendix 1).

We adopted the qualitative collective case study approach to explore the collective phenomenon of multiple HPE learners participating in a CPD course on AI technologies in HPE through a qualitative interpretative lens on multiple varied artifacts from the course and after the course [26]. This in-depth approach allowed us to understand how and why changes in perceptions, affects, and behaviors occurred due to learning about AI technologies and participating in course activities. Limitations of this approach include generalizability beyond our institution and in other educational formats, as well as smaller sample sizes.

The authors used the SRQR (Standards for Reporting Qualitative Research) checklist in reporting the development and documentation of this study (Checklist 1) [27].

### Course Design and Structure

HPE 912 was designed to equip health care educators and leaders with the knowledge, skills, and perspectives needed to integrate AI into HPE. The course was developed as a practical CPD experience that moved learners beyond conceptual awareness toward applied use of AI in authentic educational settings.

The course followed a progressive experiential arc (Multimedia Appendix 1). In the early weeks, learners explored foundational AI concepts and compared the performance of different large language models across educational tasks. In the middle portion of the course, they engaged with multimodal and generative artificial intelligence (GenAI) tools to create and revise educational products, including instructional materials and medical imagery. In the final weeks, learners examined leadership, implementation, and ethics questions related to AI adoption in their own professional contexts.

The educational design emphasized active experimentation, critical reflection, and contextual application. Each assignment required learners not only to use AI tools but also to reflect on their prompts, evaluate tool limitations such as hallucinations and bias, and justify the role of human judgment in refining outputs. In this way, the course encouraged participants to develop practical competence with common AI tools while also examining how their perceptions of the technology evolved and how AI might influence their future work as educators and leaders.

### Data Collection

#### Overview

To understand the educational strategies that were most effective in fostering long-term change in the learners and the influence of the course on the students’ practice, we employed methodological rigor by gathering data from three different sources: (1) precourse survey, (2) five written course assignments, and (3) semistructured interviews.

#### Precourse Survey

A precourse survey was administered to capture participants’ baseline perspectives on AI and their expectations for the course. The instrument (Multimedia Appendix 2) included two closed-choice items on participants’ emotional responses toward AI (eg, excitement, enthusiasm, and apprehension) and their prior knowledge and experience with AI tools. These included open-ended response items for participants to share their motivations for enrolling in the course. Participants were also asked to indicate any concerns they had about the course, the broader topic of AI, and the specific areas of AI in HPE they were most interested in exploring. This survey served to contextualize learners’ starting points and to inform course design.

This closed survey was administered online via Google Forms (Google LLC, 2024) through the course learning management system, only to the learners in the course. This was a mandatory survey that needed to be completed as part of the course requirements [28].

#### Course Assignments

Student assignments were included as a key data source to examine how participants engaged with and applied course content in authentic tasks. The assignments required learners to analyze AI models, experiment with GenAI tools, design educational resources, and reflect on leadership and implementation strategies in their own contexts (Multimedia Appendix 1). As these tasks moved beyond knowledge recall to application, synthesis, and reflection, they provided a rich window into how participants made sense of AI concepts and integrated them into their professional roles.

Analyzing these products allowed us to assess not only immediate understanding but also evidence of higher-order thinking, creativity, and transfer of learning. Moreover, the assignments, providing a naturalistic record of learners’ decision-making, challenges, and evolving perspectives, were an essential complement to survey and interview data for evaluating the course’s impact on long-term professional practice.

#### Semistructured Interviews

We conducted 45‐60-minute, semistructured interviews six months after course completion to assess the transfer of learning beyond the immediate course period. This delayed timing allowed us to explore how learners had sustained, adapted, or applied their learning in real-world professional contexts, including educational, administrative, research, and clinical practice (eg, self-reported Kirkpatrick level 3 behavioral changes and expanding connectivism learning networks). The ten semistructured interview questions focused on the research aim and question, including post-course application of learning (Multimedia Appendix 3).

We sent emails to all first cohort participants (nine learners), using IRB-approved recruitment emails that briefly described the interview purpose and research study. Only learners from the first cohort were invited for the interviews as they met the required longitudinal distance of six months since completion of the course.

Each learner voluntarily attended the interview in response to recruitment emails. They were also informed at the beginning of the interview of the option to end the session at any point. At the start of every interview, JGP reviewed the consent process with the participant and clarified the (1) purpose and nature of the interview; (2) voluntary nature of the interview and option to opt-out; (3) maintenance of confidentiality through deidentification and secure storage of interview content; (4) recording of the interviews for auto-transcription and recall purposes, but that it could be deleted if desired; (5) risks associated with the interview; and (6) contact information for the researchers should concerns arise.

Privacy associated with the interviews was achieved through virtual interviews conducted by JGP in a quiet, private office. Interviews were conducted using Google Meet (Google LLC, 2024) with the transcription feature enabled. JGP reviewed the transcripts and recordings for accuracy and clarifications or corrections.

### Data Analysis

#### Data Procedures and Technological Support

All data sources, survey responses, assignments, and interview transcripts were deidentified by a research assistant and assigned a pseudonym. The data were secured as Google Docs (Google LLC, 2024) files on the USU Google secure server. Members of the team accessed the files on encrypted password-protected computers. This deidentified data was analyzed by the researchers.

**Figure 1.**
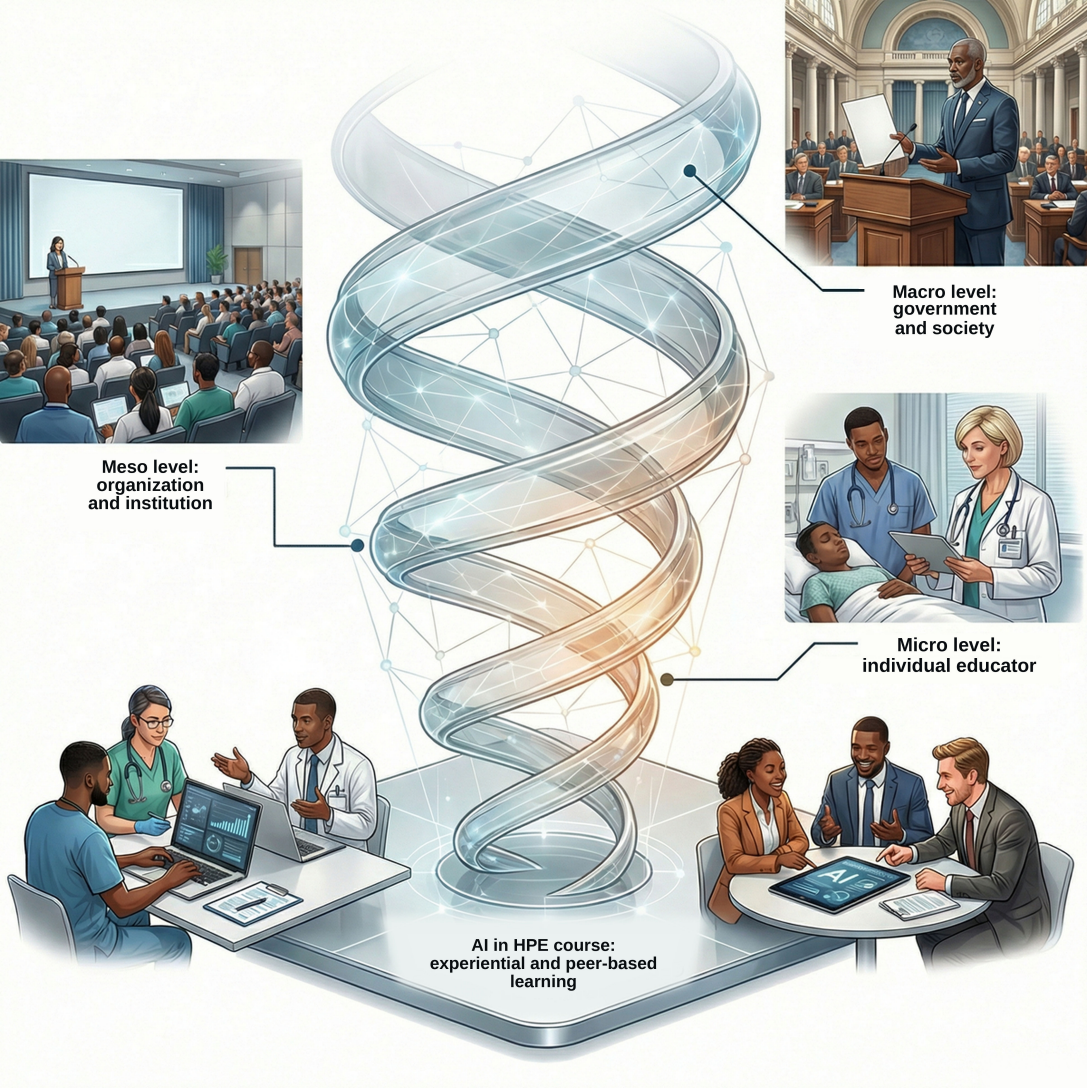
Participants in an AI-focused HPE course, grounded in experiential and peer-based learning principles (base of the helical spiral), demonstrated cyclic growth and ever-widening networks of interconnectivity (widening helix with interconnected nodes). Many learners exhibited shifts in perception, affect, and behavior at the individual (micro) level, with some demonstrating initiatives at the organizational and institutional (meso) levels, and at least one learner approaching societal and governmental (macro) levels (level-based impacts are depicted as call-out scenes). Google Gemini (Google LLC), NotebookLM (Pro version, accessed February 19, 2026), and ChatGPT DALL-E-3, OpenAI; June 10, 2026 were used to cocreate this image as detailed in Multimedia Appendix 4. AI: artificial intelligence; HPE: health professions education.

The researchers used ATLAS.ti (ATLAS.ti Scientific Software Development GmbH; 2024) to track codes and quotations. The authors acknowledge limited use of multimodal GenAI tools for two discrete tasks, as outlined in Multimedia Appendix 4. The researchers used OpenAI ChatGPT-4o (paid version) to cocreate the subthemes from the researcher-identified codes. JGP used Google NotebookLM (Google LLC and DALL-E-3, Open AI) to cocreate Figure 1, using the manuscript as the source data with author-provided meta-prompting.

#### Precourse Survey

The researchers aggregated the survey data into a Google Sheet (Google LLC, 2024) for analysis. The researchers conducted a simple descriptive analysis of the data for the two close-response questions and the open-ended question: How and what AI technologies have you used? The researchers treated the responses to the other three open-ended questions as textual data and thematically analyzed them using the thematic analysis process by Braun and Clarke.

#### Course Assignments and Interviews

The researchers treated the course assignments and interview transcripts as textual data and thematically analyzed them using the 6-step thematic analysis process by Braun and Clarke. In step 1 of the analysis, authors JGP and JM reviewed the data for familiarization and identified initial patterns within the data. In step 2, JGP and JM generated the initial codes (Table 1). JGP and JM then further refined the codes. JGP, JM, and AS generated, reviewed, and refined the themes in steps 3, 4, and 5 (Table 1). The research team addressed all discrepancies through discussion until they reached consensus. Finally, we selected compelling extracts that were rechecked against the themes and research question. Table 1 details our movement from developing codes, which the authors combined into subthemes, and then themes. This approach was especially effective because it allowed us to follow the thread and gain a deeper understanding of the relationships between these elements [29].

**Table 1.** Themes, subthemes, and codes.

Themes	Subthemes	Codes
AI^a^ misconceptions breed fear	Navigating initial encounters with AI	Embarrassed Initial misconception about AI use Initial response to AI Intrigued but resistant Precourse AI misconceptions Precourse perceptions of novice status
Education leads to a shift in perspective	Optimizing the learning experience shifting perspectives and addressing concerns	Changes in learning/education Creativity Free experimentation Hands-on practice to learn AI Learning through play Repetition produces familiarity Teacher-learner interaction AI fears/concerns Hope in future Postcourse changed perspective Postcourse understanding of ethics Professional identity Trust/confidence Freedom/flexibility Various modalities enhanced learning Modeling
As perspectives shift, learners reach out to their social circles	Developing competence and inspiring action	Need for further education Postcourse AI initiatives Postcourse motivation Postcourse perception of abilities Perceived benefits of AI Postcourse AI limitations
Confidence in AI technologies leads to implementation and advocacy	Fostering knowledge dissemination and advocacy	Demonstrations of AI advocacy Sharing knowledge with peers/friends/family Institutional barriers Applicability to real world

a AI: artificial intelligence.

#### Data Analysis Summary

Unlike the survey and assignments, which captured immediate reactions and behavioral applications within course boundaries, the interviews provided insight into longer-term engagement, barriers to implementation, and the integration of AI tools into practice. By focusing on learners’ reflections after an extended interval, these interviews offered a critical perspective on the durability and impact of CPD in fostering meaningful change. Together, these three data sources provided a comprehensive view of the learners’ journey from entry into the course through to longer-term professional application. This multimethod approach allowed for a richer understanding of how CPD in AI influenced Kirkpatrick Level 1‐3 learning in written and oral samples.

To establish the rigor of our findings, we intentionally transitioned away from the positivist concept of data saturation, which is frequently incompatible with interpretivist research. Instead, guided by recent frameworks for assessing qualitative data adequacy [30] and the conceptualization of information power by LaDonna et al [31], the researchers evaluated data adequacy based on the specific characteristics of this study’s design and the resulting artifacts. Sufficiency was assessed iteratively during the analysis. As this study possessed a narrowly focused research question, a highly specific participant sample (educators who completed this exact experiential course), and interview dialogue characterized by rich conceptual depth, the dataset was deemed to have high information power [31]. Through this lens, the research team determined that 105 written assignments from 21 participants and 5 in-depth interviews achieved the theoretical sufficiency needed to address the exploratory research aims. In alignment with this framework, we prioritized the richness, resonance, and conceptual depth of the data over absolute sample size.

By using the experiential learning theory by Kolb as an a priori theoretical lens, we sharpened our analytical focus and enhanced the evidentiary value of this study. This iterative analytical process allowed us to move beyond surface-level descriptions, reaching a point of analytical sufficiency where the findings offer a robust, conceptually thoughtful account of how hands-on experimentation and reflective practice dismantle precourse misconceptions, ultimately fostering the confidence and trust required for institutional AI implementation.

#### Researcher Characteristics and Reflexivity Statements

JGP is a nuclear radiologist passionate about AI technologies for educational and clinical practice. He believes AI tools can help educators be more effective and efficient in their teaching efforts, and that these tools, when used ethically and appropriately, can increase creativity and critical thought about complex problems. His views are reflected in the scope, development, and reporting of this study. He helped in the initial development of the first HPE course, but he did not act as an official instructor or grader for either course to reduce research bias and prevent undue influence during the interviews. He performed the interviews described below and participated as one of the coders during the analysis. To reduce bias, he did not review the presurvey or course assignments until independent coding analysis began. Through open-ended, semistructured interview questions, he attempted to mitigate personal influence on the interviews.

JM is an educational psychologist who uses mixed-methods research to explore metacognition and emotion in medical education. Her expertise fundamentally shaped this study’s methodology, analysis, and interpretation to prioritize participants’ metacognitive reflections and emotional responses to AI technologies. She participated as an independent coder during the analysis. She was not involved in the course in any way and only participated in the research phases of this study.

AS is an online educator and researcher. Her research straddles the domains of adult learning, online learning, and health education. We recognize our views have a critical implication on the ethical and effective integration of AI in educational settings. She developed the HPE course and was the primary instructor and grader for both course cohorts. To reduce bias, she did not participate in data collection or initial coding. She participated in a subsequent thematic analysis to avoid influencing the findings of this study.

## Results

### Participant Demographics

The researchers included survey data and assignments from 21 learners in two course cohorts for this study. Study participants ranged in age from 26 to 54 years with an average age of 42 years. This study included 10 male and 11 female participants. Only 14 (67%) of the participants were physicians, with the others spanning specialties from dentistry, nursing, pharmacy, laboratory sciences, and physical therapy. The cohorts included 2 medical student educators. The researchers collected written artifacts from 21 learners in two cohorts of the course, including spring 2024 and 2025 offerings.

A total of 5 of the 8 (62.5%) learners in the first cohort (1, 2, 4, 6, and 8) responded to the IRB-approved survey recruitment email and volunteered to participate in the interview. We noted that the nonresponding learners consisted of 2 surgeons and 1 resident physician. The 5 respondents consisted of 2 family medicine physicians (1 in administration/clinical practice), 1 pediatric physician, 1 surgeon (administration/clinical practice), and 1 laboratory supervisor.

### Study Findings

In this study, we explored how a CPD course for HPE professionals on AI technologies could influence the perceptions, effects, and behaviors about AI technologies. Our findings revealed that AI misconceptions breed fear toward AI technologies and that experiential and peer-based education can lead to a shift in perspective. In turn, this leads HPE learners to influence their personal social circles. For some learners, confidence in the use of AI technologies led to increased self-reported implementation and advocacy for AI in their organizations. Table 1 summarizes the aforementioned codes, subthemes, and themes identified by the researchers through inductive analysis of the learners’ artifacts, including presurvey, written assignments, and semistructured interviews [32]. We report selected quotes, aligning with these themes, in the text below. Supplemental quotes supporting these themes can be found in a supplemental table (Multimedia Appendix 5).

### Precourse Survey

#### Apprehensive, but Curious, Learners Engaged in the Course

Overall, 15 of 21 (71%) respondents for the precourse survey noted feeling apprehension about the use of AI, and 14% (n=3) mentioned fear. These feelings support the identified codes aligning with the “AI misconceptions breed fear” theme (Table 1). A total of 52% (n=11) and 67% (n=14) of respondents identified feeling excitement and enthusiasm for the use of AI, respectively. Only 10% (n=2) of participants reported having had no exposure to AI before the course. Few learners (n=1) in the first cohort reported having used AI in some capacity, compared with the second cohort (n=9), resulting in a total of 10 (48%) participants for both cohorts. An additional 39% (n=9) of learners in both cohorts reported basic knowledge about AI. The course was an elective; therefore, participants were interested in AI and curious about it when they enrolled in the course.

In their precourse questionnaire, 2 interviewees reported no prior knowledge of AI, 2 interviewees reported basic limited AI knowledge, and 1 interviewee reported some experience with using AI tools or applications. All demonstrated some enthusiasm or excitement about AI, but 3 interviewees also reported apprehension or uncertainty (Table 1). All reported taking the course to learn more about AI technologies and how they could be used for health care and education, with P6 describing his motivation as “options are either to hide my head in the sand and become obsolete, or develop proficiency and continue to grow along with the practice of medicine.”

### Reflective Assignments (A) and Interviews (I)

#### AI Misconceptions Breed Fear

Universally, learners remarked on the misconceptions that they had about AI before the course (Table 1). P19 (A) expressed it best as, “if I had been asked frankly what I perceived AI to be, my answer was often drawn to 30-year-old movies like Terminator or Maximum Overdrive.” Misconceptions included the idea that using AI in education and practice was unethical, that AI tools were infallible, that AI would replace them as physicians or educators, that AI would not be useful in academic or clinical practice, and that they were not currently using AI in their daily lives. As P8 (I) stated, “I was so afraid of the ethical dilemmas like ‘Oh my God, if these words aren’t mine, is that okay?’” These misconceptions about AI lead learners to fear AI and avoid its use in their personal and professional lives.

Learners also expressed fears that “the potential risks…of AI in medical education are limitless. The dangers of plagiarism, hallucinations, and bias are worrisome” (P10 (A)). As expected of health professions educators, many expressed fears about learning and education, noting that “trainees would use it to complete assignments, personal statements, etc, essentially making these ‘useless’ in evaluation” (P1 (A)). The learners described AI fears about maintaining work originality, inaccuracies, or “hallucinations,” “deep fakes,” or other nefarious uses of AI technologies, patient safety, bias, inability to keep up with the pace of AI technologies, changes to their professional practice or losing their jobs, an inability to learn something new or technologically challenging, and a fear of the unknown.

#### Education Leads to a Shift in Perspective

Education, however, was able to create a shift in the learners’ perspectives (Table 1). Many learners described that their initial fears and misconceptions were reduced or eliminated due to attending the course: “I’m like, ‘God, I have to engage’ and I’m like, ‘How do I do this?’ And so, it really helped me sort of start my journey” (P2 (I)). As learners engaged experientially with the content in the course, they began to recognize the misconceptions they had previously held about AI technologies. One of the first realizations for many learners was that AI tools are integrated into almost every aspect of modern life. P1 (A) clearly articulated “I erroneously thought that I had almost no interaction with AI…I was surprised to learn that I interacted with AI on a daily basis…”

Many remarked how they would not have imagined that they would have used AI technologies before the course as an expert health care educator, but as they worked hands-on with the tools that had quickly changed. For instance, as P13 (A) reported, as “I reflect on my journey through this course on artificial intelligence (AI) in health professions education, I recognize the significant evolution in both my understanding of AI and my enthusiasm for integrating it into my professional endeavors.” They also realized the imperfections, hallucinations, and errors that the tools produced. For example, participating firsthand in the image creation activities, they realized that AI tools could not draw a perfect hand or an image with text on it. As participant P7 (A) stated, “one interesting thing is that the AI has a lot of trouble with hands…”

Participants actively discussed ethical concerns with faculty, guest lecturers, and peers, and began to describe a shift in understanding about the way they perceived ethical uses of AI in their daily lives, academic work, and clinical practice. It shifted from a concrete view of ethics to a more nuanced perspective on where, when, and how AI tools could be used. Furthermore, many described how their concerns of naivete about AI technologies caused them to be hesitant to learn or use the tools.

They described how the class provided opportunities to remove self-imposed limitations to their use, bravery to use the tools, and the realization that they were in a similar position of novice status relative to their peers and other health care professionals. Learner P6 (I) stated that AI technologies “don’t require other people, they just require me to be brave enough to create an account and yeah, screw up…” Importantly, many learners described a change in their affect and emotional response to AI technologies. The AI fears described before the class gave way to emotions of excitement, enjoyment, and hope about AI technologies and their potential to improve their lives and careers as health care professionals and educators. For instance, P14 (A) noted, “I think the biggest surprise was how much this course served as a paradigm shift for me. How does an entire healthcare system or medical education program shift from what amounts to written rules to algorithms and generative ways of proceeding?” As learners’ perspectives and emotions around AI shifted, they described how they would reach out to their social circles to share, teach, and use their newly developed knowledge about AI.

#### Influencing Personal and Social Circles as Perspectives Shift

This excitement in the AI tools shifted to a natural tendency to share that knowledge with their social circles in peer-based approaches (Table 1). P19 (A) described this excitement about sharing with their social circles as follows, “I met each assignment with such excitement and shared my new knowledge with my family and my colleagues at work…I became a weekly feature on our team huddles, sharing my latest knowledge and providing information on how it could be sourced in an organization that was not currently set up for the use of AI.” For many, the closest social circle was their own family members. Learners described teaching their children about the ethical and effective use of AI tools for their schoolwork. They shared how they were able to appropriately modify their response to their children’s use of AI through a more nuanced understanding of the ethical uses of AI. For some individuals, their family members were more knowledgeable about AI technologies, and so they would reach out to learn more from them.

Additionally, learners discussed how they would learn from their peers in the class. They expressed how it was good to brainstorm ideas, discuss challenges, and develop new uses for the AI tools discussed in class. P7 (A) stated that “I certainly was impacted by peer learning and just seeing and reading about some of what my peers were doing with AI.” Learners also described peer-based learning with their trainees. They shared how their own health care trainees would teach them about new AI technologies, and how they would instruct them on effective and ethical AI tool use. In describing this experience, P18 (A) noted, “I believe student collaboration in particular will help me stay abreast of AI development since the younger learners are extremely forward leaning in that direction.” As a component of this social circle-based learning, some learners described the use of social media in helping to develop more knowledge about AI tools. The socialized learning of AI technologies among the learners, their families, and their peers led to a natural progression of technology implementation in their professional careers, development of programmatic and institutional education, and advocacy for AI technologies in their spheres of influence.

#### Confidence in AI Technologies Leads to Implementation and Advocacy

As learners learned about, experimented with, and used the AI tools in class, they began to implement the AI tools in their daily lives (Table 1). Many reported initially using the tools for simple administrative tasks, such as writing emails and planning vacations. As educators, they also turned to the implementation of AI tools in basic educational activities, such as building curricula, summarizing clinical information, revising curriculum vitae, developing clinical templates, writing questions, generating learning objectives, developing patient scenarios for education, and developing a second opinion. As P1 (A) explained, “The utility of these within my role is almost endless… from assisting me with drafting initial educational components more efficiently to generating images for educational presentations to more effectively drafting communications I send to staff (ie, emails, newsletters, etc).”

Many learners described how the AI tools helped to reduce the cognitive load and time spent developing and crafting a project or product. They described how AI tools helped reduce the blank page anxiety that comes with starting a new project. As P6 (I) stated, “It was really useful for that, overcoming, what, Picasso described as the tyranny of the blank canvas. Going from ‘How do I even get started on this’ to say ‘Okay, I’ve got a block architecture. I can modify that as needed.’” In reducing the activation barrier for learners, they were able to begin new endeavors more confidently. They noticed how the AI tools made their jobs easier and time-efficient.

P8 (I) described a particularly impactful experience with not knowing what to say or do for a learner who was struggling with mental health concerns. The learner used AI tools to help craft an appropriate message, “we had a resident who was having a really rough time with mental health and I wanted to send them a message and I wasn’t sure of the right words to say in this situation…it was super helpful just helping me come up with a nice message to send.”

Some learners began to develop more complex applications of the AI tools that impacted more than just themselves and their small social circles. They worked with others to develop educational courses, curricula, and tools for clerkship rotations. P21 (A) reported one such presentation: “I recently developed and delivered a grand rounds on the topic of AI. The focus of this presentation was on the evolution of AI, evolution of medicine, and current AI being used within [my organization].” With increasing understanding, confidence, and real-world application of the AI tools, learners felt more empowered to begin advocating for AI technologies in their programs and institutions. Many acknowledged that there were challenges with implementing AI tools in their institutions and in health care practice, but they still sought ways to promote knowledge of AI technologies, the ethics of AI, and the application of AI technologies in daily practice.

Despite institutional challenges, P1 (A and I) described developing and organizing an entire day devoted to discussing the application of AI technologies in education and clinical practice. She worked with AI experts at her institution and educational leaders to build support and invited speakers to come speak about their work with AI technologies. P8 (I) described how she worked with fellow medical student site directors to develop a specific AI tool that would provide improved, targeted, narrative feedback for medical students working on specific entrustable professional activities. These were then distributed to medical student sites nationwide. P6 worked with AI experts at his institution to develop workshops centered on learning about AI technologies and how they could be used in clinical practice.

A summary of these educational advocacy approaches was best conceptualized as follows:

*By integrating AI into my teaching and research, I can help my students and colleagues better understand its potential benefits and encourage a more open-minded approach to its use…This has been significant because it has helped me recognize that I can not only use these tools but also advocate for their use in educational settings. As an educator, this is empowering—it allows me to introduce AI to my learners and help them see how they can use these tools to enhance their own careers.*

P13 (A)

The AI course empowered some educators to learn, explore, and experience AI tools, which they shared socially, building communities that impacted their programs and institutions. As learner P12 (A) indicated, “we all must become AI leaders…”

## Discussion

### Summary of Findings

In our study, we found that experiential, practical, and peer-based learning methods were effective in helping HPE learners learn how to effectively use AI tools in their daily activities. As learners began using AI tools in their everyday lives, they reported a decrease in previously held misconceptions and fears about AI technologies (Table 1). They began to explore other areas for AI tool integration and application in their personal and professional lives. Learners learned about new AI tools from their family members, peers, and students. In turn, they also reported teaching others about what they learned from the course in increasingly wider spheres of influence. Learners reported their use of AI tools from the class in educational, administrative, and research settings. Ultimately, the course influenced learners in micro, meso, and macro contexts within their personal and professional lives (Figure 1) [10,10,33].

### Precourse Fears and Misconceptions

Many HPE learners in our CPD course had misconceptions about AI technologies that often led to apprehension, fear, and a tendency to avoid its use. These beliefs included the idea that integrating AI into education and practice is inherently unethical, that AI tools are without flaws, and that AI will inevitably replace human professionals. What does it mean to be a health care professional working with a tool that is smarter than you? What does it mean for professionals using these tools in clinical practice? Furthermore, learners frequently underestimate how much they already interact with AI in their daily lives and its potential value in both academic and clinical settings. Prior research confirms that this general unease is a common psychological barrier to engaging with new technologies [25,34].

Beyond these misconceptions, specific emotional anxieties were prevalent. Health care professionals, for instance, voiced concerns about AI’s impact on learning and evaluation, fearing that trainees might use AI to complete assignments, thereby diminishing the integrity of traditional assessment methods. Ethical considerations surrounding AI, such as the authenticity of AI-generated content, also caused considerable worry. Other fears encompassed the risks of plagiarism, AI-generated inaccuracies, and bias, alongside worries about patient safety, the rapid advancement of technology, potential job displacement, and feelings of personal inadequacy in learning new, technologically complex skills. Hosseini et al [35] and others have also echoed that these fears present challenges for health care professionals in adopting AI technologies. Collectively, these fears suggest an apprehension may stem from a lack of understanding and direct experience with AI tools in their daily practice.

### Postcourse Shift in Perspective and Engagement

Post-course interviews with 5 HPE learners from the first cohort demonstrated a consistent shift in perspective through direct engagement with AI tools, manifesting across micro, meso, and macro levels, achieving self-reported Kirkpatrick level 3 outcomes [18,33]. This evolution of ever-widening spheres of peer influence and knowledge acquisition aligns with connectivism, a learning theory that views knowledge as a process of navigating nodal networks composed of both human and technological sources [23,24]. In the context of rapidly advancing AI, these networks require HPE learners to engage in a continuous cycle of integrating new nodes—such as emerging platforms, technical expertise, and diverse peer groups—which fosters a critical metacognitive ability to evaluate and prioritize information. By facilitating experimentation through discussions and peer-based learning, faculty enabled participants to build and maintain these expanding networks, effectively shifting their educational perspective from isolated skill acquisition to a systemic, networked understanding of AI integration within their professional environments [33].

### Micro Level

Crucially, our case study demonstrates that direct engagement with AI content through structured, hands-on, experiential education can significantly alleviate or eliminate personal fears and misconceptions [36]. In line with experiential learning theory, we found that the ability for learners to experiment with the AI tools, practice their AI skills, and even fail in their desired outcomes (in a safe environment) may help learners to more rapidly shift their perspectives regarding AI technologies [22]. This shift in perspective often began with the self-reported, “surprising” realization that AI is far more integrated into daily life than previously understood. Learners initially believed they had minimal interaction with AI, only to discover its pervasive presence in their routines. This newfound awareness was an important initial step in demystifying AI technologies and fostering a more receptive mindset.

Moreover, direct interaction with AI tools enabled learners to recognize their imperfections and limitations, such as AI’s occasional difficulty in accurately rendering hands or text in images. Merkebu and Samuel [25] also highlight that “using customizable and psychologically safe strategies, effectively dismantles globally shared anxieties, such as the fear of inadequacy, making AI adoption feasible and empowering individuals across diverse contexts.” This experience challenged the notion of AI infallibility and fostered a more realistic understanding of its capabilities. Discussions with faculty, guest lecturers, and peers on ethical considerations also facilitated a transition from a rigid, black-and-white view of AI ethics to a more nuanced and context-dependent understanding of its appropriate use. This evolving perspective highlighted that many professionals in our course are still navigating how and where AI can effectively fit within their practice.

The educational experience may also help learners overcome their cognitive and meta-cognitive limitations and embrace a willingness to experiment with AI tools. Recognizing that many of their peers shared a similar beginner status with AI fostered a supportive learning environment. This comprehensive engagement with AI content led to a notable change in learners’ emotional responses, transforming initial feelings of fear and inadequacy into enthusiasm, enjoyment, and optimism about AI’s potential to enhance their professional and personal lives as health care professionals and educators. Extending prior works, we demonstrated that well-designed educational interventions are essential in bridging the gap between apprehension and active engagement with rapidly evolving AI technologies [25].

### Meso Level

In line with connectivism theory, we found that as many HPE learners in our course experimented with, practiced, and became more confident in their AI abilities, they began to share their knowledge and skills with others, developing new nodes in their developing learning networks [23,24]. Often, learners begin by sharing with family members and learning from those close individuals. As educators, many also began to share and learn from their peers and their students. This peer-based learning has been shown to be effective in developing clinical AI champions in stressed health care systems by Teferi et al [37]. Similarly, Zhou and Schofield [38] also argue that AI has the potential to promote collaborative and communal learning. Emotions of surprise, joy, and excitement often accompanied this desire to share and learn from others.

Extending prior research, the shift in perspectives and emotions led learners to share and learn from peers and family members about different aspects of AI technologies [39]. Parent learners often shared what they had learned about the ethical use of AI technologies with their children, which may not have been possible without the course. Adult family members and peers often provided more knowledge and expertise about topics that were at the boundaries of the learner’s expertise and experience [40]. In some cases, learners were able to share and teach about what they learned with their peers, leading to the development of interconnected nodes of learning and excitement about AI technologies, as described in connectivism learning theory. As students in our course developed these local and personal interconnected networks, they developed AI confidence and competence that allowed them to reach out to larger organizations and networks of influence [39].

### Macro Level

Notably, in our post-course interviews with some learners, we discovered that some learners expanded their networks of AI learning and knowledge to their organizations and medical communities, describing actions that serve as preliminary steps toward advocacy for AI technologies in their practices. For some learners, these ideas of advocacy were aspirational, but for others, they made efforts to implement change in their larger spheres of influence. The capacity shown by some learners to navigate complex networks and structures to develop engaging content for their peers and trainees demonstrates excitement, motivation, and commitment developed during the course. These early advocacy actions and motivations demonstrate a preliminary macro-level shift in a few HPE learners beyond their close social spheres to expanding spheres of collaboration and influence, advocating for and instituting changes in their health care systems [21].

### Limitations

Limitations of this study must be acknowledged. First, this study has been conducted with a small number of participants from one institution. Furthermore, the sample size of interview participants was also small. While this might raise questions of generalizability, it must be noted that qualitative studies aim to provide a deeper and more detailed understanding of people’s experiences [41]. Next, the participants in this study all self-selected to participate and entered the course out of curiosity and interest. While this could be seen as skewing the current study, it is interesting to note that these participants also described fear and apprehension at the start of the course. Furthermore, while our participants discussed initiatives they had started in their organizations, this is limited to self-reported data, as we did not verify the information with the organizations. Future studies could focus on change at Kirkpatrick level 4 (organizational change) and macro levels, including objectively examining participants’ actions.

### Conclusions

AI technologies are rapidly disrupting and changing many industries and professions, including health care. To address this rapidly evolving environment, it is imperative to prepare health care educators with the requisite knowledge, skills, and attributes to ethically and effectively integrate, use, and teach about AI technologies. In this study, we demonstrate how practical, experiential, and peer-based learning fosters confident and perceived competent health care educators, resulting in self-reported level 3 Kirkpatrick outcomes [18]. Some educators even reported the beginnings of broad-based advocacy for AI technologies within their institutions and broader health care communities. Excited and empowered educators share, teach, and continue to learn about the AI technologies, establishing ever-expanding and interconnected networks of learning.

## Supplementary material

10.2196/87381 Multimedia Appendix 1 Artificial intelligence in health professions education: continuing professional development course description, objectives, assignments, artificial intelligence usage policies, schedule, and grading rubrics.

10.2196/87381 Multimedia Appendix 2 Artificial intelligence in health professions education: continuing professional development course presurvey questions.

10.2196/87381 Multimedia Appendix 3 Artificial intelligence in health professions education: continuing professional development course, semistructured interview questions.

10.2196/87381 Multimedia Appendix 4 Description of the use of multimodal generative artificial intelligence tools in this paper.

10.2196/87381 Multimedia Appendix 5 Additional thematic quotes.

10.2196/87381 Checklist 1 The SRQR (Standards for Reporting Qualitative Research) checklist for qualitative research was used in the conception and reporting of this manuscript [27].

## Abbreviations

AI: artificial intelligence
CPD: continuing professional development
GenAI: generative artificial intelligence
HPE: health professions education
IRB: institutional review board
SRQR: Standards for Reporting Qualitative Research
USU: uniformed services university
