# Supplementary material for: Impact of a Practical, Hands-On, Continuing Professional Development Course About AI in Health Care Professions Education on the Perceptions and Behaviors of Health Care Educators: Qualitative Case Study
Source: JMIR Med Educ. 2026 Jun 23;12:e87381. doi: 10.2196/87381 (PMC13290435; doi:10.2196/87381)
Supplement: Multimedia Appendix 1 — Artificial intelligence in health professions education: continuing professional development course description, objectives, assignments, artificial intelligence usage policies, schedule, and grading rubrics. [file mededu-v12-e87381-s001.pdf]

Course Description

This 6-week course delves into the applications of Artificial Intelligence (AI) in healthcare practice and education. Students will explore how AI technologies can enhance teaching, learning, assessment, research, and feedback in healthcare education. Students will develop an understanding of the potential applications, challenges, and ethics in utilizing AI as leaders in healthcare education.

Course Objectives

At the completion of this course, participants will be able to:

1. Explain the fundamental concepts of AI and its potential applications in HPE.
2. Apply AI techniques, such as NLP and ML to enhancing learning in healthcare.
3. Critically evaluate the ethical implications of using AI in healthcare and education.
4. Design AI-powered learning experiences to meet the needs of healthcare professionals.
5. Develop knowledge and skills to lead out in communicating the benefits and risks of AI to healthcare stakeholders.

Assignments

This course includes 5 assignments as follows:

ASSIGNMENT	GRADING
Pre-course survey	Complete/Incomplete
Comparative analysis of AI Chat models	Exemplary/Proficient/Basic
Exploring AI-Generated Images	Exemplary/Proficient/Basic
Using Generative AI in Medical Education Design	Exemplary/Proficient/Basic
Leadership in Implementing AI	Exemplary/Proficient/Basic
Final reflection	Exemplary/Proficient/Basic

AI USAGE

Students are allowed to use advanced automated tools (artificial intelligence or machine learning tools such as ChatGPT or Dall-E 2) on assignments in this course if that use is properly documented and credited.

For this course, you will need to share the prompts you use.

ChatGPT instructions:

On your ChatGPT prompt screen, click on the upload button on the top right and follow directions.

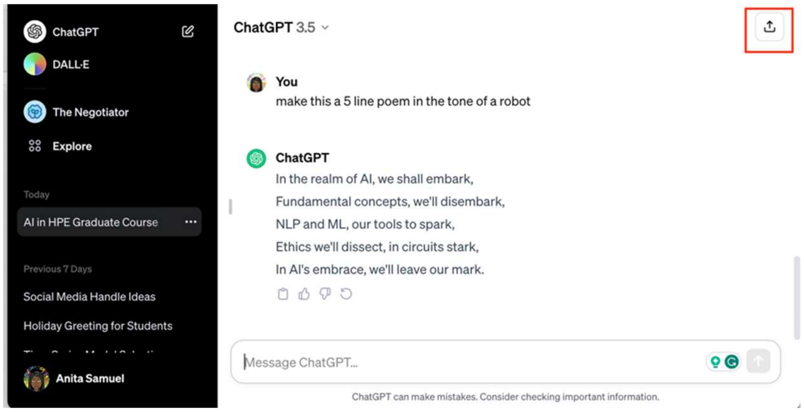

Google Bard:

Instructions at: https://support.google.com/bard/answer/13743730?hl=en&co=GENIE.Platform%3DDesktop

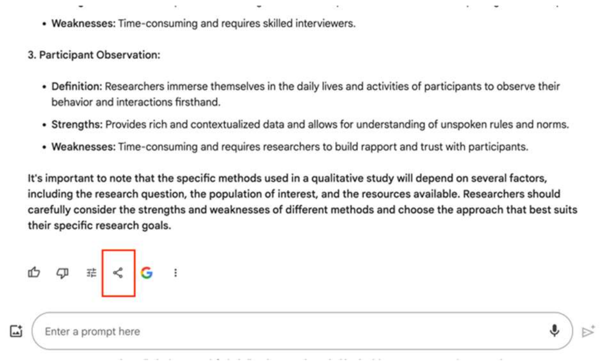

Course Schedule

NOTE: Other videos might be added to the course materials and will be available on Canvas.

Weeks	READINGS / VIDEOS	ACTIVITIES
Week 1<br>Introduction to AI<br>(Jan 2-7)	Readings:<br>• Artificial Intelligence for Health Professions Educators<br>• Artificial Intelligence in Medicine: Today and Tomorrow<br>• Cooper A, Rodman A. AI and medical education—a 21st-century Pandora's box. N Engl J Med. 2023 Aug 3;389(5):385-7.<br><br>Watch:<br>• What AI is…and isn't [24.12]<br>• 7 types of AI<br><br>Listen:<br>• Interview with Adam Rodman on the potential effects of generative artificial intelligence on medical education and clinical practice	By 1/7:<br>• Pre-course survey
Week 2<br>(Jan 8-14)<br>Large language models	Read:<br>• 100+ ChatGPT prompts for healthcare professionals<br>• Meskó, B. (2023). Prompt engineering as an important emerging skill for medical professionals: tutorial. Journal of Medical Internet Research, 25, e50638.<br><br>Watch:<br>• How Large Language Models Work<br>• Why large language models hallucinate?	By 1/14:<br>• Comparative analysis of AI Chat models
Week 3<br>(Jan 15-21)<br>Image and Video Creation & Analysis	Read:<br>• Huston JC, Kaminski N. A Picture Worth a Thousand Words, Created with One Sentence: Using Artificial Intelligence–created Art to Enhance Medical Education. ATS Scholar. 2023 May:ats-scholar.<br>• Ahmad Z, Rahim S, Zubair M, Abdul-Ghafar J. Artificial intelligence (AI) in medicine, current applications and future role with special emphasis on its potential and promise in pathology: present and future impact, obstacles including costs and acceptance	By 1/21:<br>• AI image generation

	among pathologists, practical and philosophical considerations. A comprehensive review. Diagnostic pathology. 2021 Dec;16:1-6.<br><br>Optional reading:<br>• Efimenko M, Ignatev A, Koshechkin K. Review of medical image recognition technologies to detect melanomas using neural networks. BMC bioinformatics. 2020 Sep;21:1-7.<br>• Fourcade A, Khonsari RH. Deep learning in medical image analysis: A third eye for doctors. Journal of stomatology, oral and maxillofacial surgery. 2019 Sep 1;120(4):279-88.<br><br>Watch:<br>• How AI Could Change the Future of Medicine<br>• AI art, explained<br>• The Best AI Art Generators (Free & Paid)	
Week 4<br>(Jan 22-28)<br>AI-enhanced learning content creation	Read:<br>• Accelerating medical education with ChatGPT: an implementation guide<br>• Hosseini M, Gao CA, Liebovitz DM, Carvalho AM, Ahmad FS, Luo Y, MacDonald N, Holmes KL, Kho A. An exploratory survey about using ChatGPT in education, healthcare, and research. medRxiv. 2023:2023-03.<br>• Montenegro-Rueda M, Fernández-Cerero J, Fernández-Batanero JM, López-Meneses E. Impact of the implementation of ChatGPT in education: A systematic review. Computers. 2023 Jul 29;12(8):153.	By 1/28:<br>• Using Generative AI in Medical Education Design
Week 5<br>(Jan 29-Feb 4)<br>Leadership Implications	Read:<br>• Watson, G. J., Desouza, K. C., Ribiere, V. M., & Lindič, J. (2021). Will AI ever sit at the C-suite table? The future of senior leadership. Business Horizons, 64(4), 465-474.<br>• Peifer, Y., Jeske, T., & Hille, S. (2022). Artificial intelligence and its impact on leaders and leadership. Procedia Computer Science, 200, 1024-1030.<br>• Quaquebeke, N. V., & Gerpott, F. H. (2023). The Now, New, and Next of Digital Leadership: How Artificial Intelligence (AI) Will Take Over and Change Leadership as We Know It. Journal of Leadership & Organizational Studies, 15480518231181731.	By 2/4:<br>• Leadership in Implementing AI

	Optional Readings<br>• Daye, D., Wiggins, W. F., Lungren, M. P., Alkasab, T., Kottler, N., Allen, B., ... & Langlotz, C. P. (2022). Implementation of clinical artificial intelligence in radiology: who decides and how?. Radiology, 305(3), 555-563.	
Week 6<br>(Feb 5-9)<br>Wrap Up	Watch:<br>• 10 AI tools to try<br><br>Listen:<br>• Interview with Adam Rodman on the potential effects of generative artificial intelligence on medical education and clinical practice	By 2/9:<br>• Final reflection

Assignment Descriptions

1. Pre-Course Survey

**Purpose of the Assignment**	To gauge students' baseline knowledge, motivations, and concerns regarding AI in health professions education.

2. Comparative Analysis of AI Chat models

**Purpose of the Assignment**	To understand the capabilities, nuances, and potential applications of different generative LLM AI models—ChatGPT, Bard, and Claude—in health professions education.<br><br>As technology becomes more integrated into healthcare, it's crucial for health professionals to be familiar with and understand the capabilities of tools like AI. Incorporating them into education prepares students for future technological advancements in the field.<br><br>By analyzing different models like ChatGPT, Bard, and Claude, you can understand the strengths and weaknesses of each, ensuring the most suitable model is utilized for specific educational needs.

Due Date: 1/14

Instructions:

1. Setup & Exploration:
   - Register or access the respective platforms that host ChatGPT, Bard, and Claude (or another platform you want to try).
   - Familiarize yourself with the basic functions of each chatbot by engaging in general conversations with them.
2. Scenario Creation:
   - Design two different HPE-related scenarios that you'd like to explore with each chatbot. These could include:
     - Answering a complex medical question.
     - Discussing the ethics of a particular medical procedure.
     - Seeking clarification on medical terminology or concepts.
3. Interaction & Documentation:
   - Engage each chatbot (ChatGPT, Bard, and Claude) using the scenarios you've designed.
   - Document each chatbot's responses to the scenarios. Copy the prompt/s and responses of each tool and include it in your document.
4. Analysis & Reflection:
   - Compare and contrast the responses from ChatGPT, Bard, and Claude for each scenario.
   - Reflect on the potential uses of these chatbots in medical education. Consider their strengths, limitations, and areas of improvement.
   - Capture nuances, depth of answers, accuracy, and any other relevant observations.

Grading Rubric:
Your participation and posts will be evaluated based on:

	**Exemplary** This represents the highest level of mastery. Learners not only meet but often exceed the expected standards. Their performance serves as a model for others.	**Proficient** Learners consistently meet the expected standards. While there's room for growth and enhancement, their performance is satisfactory and competent.	**Basic** This level indicates that performance is below the expected standard. Additional training, guidance, or resources might be required.
**Depth of Exploration:** How thoroughly you've engaged with and explored each chatbot.			
**Analysis Quality:** Depth, clarity, and insightfulness of your comparative analysis.			
**Report Presentation:** Structure, coherence, and presentation of your report.			
**Reflection:** Thoughtfulness in considering the potential applications and implications of using these chatbots in medical education.			

3. Exploring AI-Generated Images

**Purpose of the Assignment**	To understand and apply AI technology in the creation of visually distinct images using an AI image generation tool.

Due Date: 1/21

Instructions:

Image Creation

- Think of an image you want to generate
- Use an AI image generation tool to create the image
- Explore the AI's capabilities by varying prompts and settings to see how minor changes affect the output.

Reflection

- Analyze the AI-generated images in terms of their adherence to your prompt
- Reflect on the creative process, noting any challenges faced and how the AI's interpretations differed from traditional human-created art.

Deliverables:

- Submit a document with at least 3 variations of the image, the prompts used and address the reflection prompts above.

Assessment Criteria:

Creativity and originality in the use of AI for image generation.
Insightfulness in the analysis and reflection on the AI-generated artwork.

4. Using Generative AI in Medical Education Design

**Purpose of the Assignment**	To leverage generative AI in creating or redesigning an educational component—be it course objectives, assignments, or other learning materials—in medical education.<br><br>This assignment encourages you to think critically about the integration of AI in educational design. While generative AI can be a powerful tool, always consider the needs of learners and the goals of the curriculum when crafting or refining materials.

Due Date: 1/28

Instruction:

1. Selection of Educational Component:
   - Choose an educational component you'd like to create or redesign. This could be anything including:
     - Course objectives for a new topic.
     - Assignments or assessment tasks.
     - Study guides or supplementary learning materials.
2. Select the Generative AI tool:
   - You have tried 3 tools in assignment 3. You can select any one of these tools or another one.
3. Utilize Generative AI:
   - Use the generative AI platform/tool to assist you in creating or redesigning your chosen educational component. This could involve:
     - Generating potential course objectives.
     - Drafting assignment prompts or questions.
     - Creating summaries, flashcards, or other learning aids.
   - Once you've generated content, refine it. Remember, while AI can produce valuable outputs, it often requires a human touch for context, relevance, and clarity.
4. Documentation & Reflection:
   - Compile a document that includes:
     - The original version (if it's a redesign).
     - The AI-generated version.
     - Your refined and final version.
   - Reflect on the process:
     - How did generative AI aid in the creation/redesign?
     - What challenges did you face?
     - In what scenarios might you consider using generative AI in the future for educational design?

Grading Rubric:
Your participation and posts will be evaluated based on:

	Exemplary	Proficient	Basic
**Creativity & Innovation**	Demonstrates a high degree of originality and innovative use of generative AI.	Shows creativity and some innovative use of AI, but may be somewhat conventional in places.	Uses AI in basic ways; limited creativity in the educational component.
**Refinement & Personal Touch**	Content is thoroughly refined, demonstrating deep understanding and context. Personal touch is evident throughout.	Content shows good refinement, with areas of personal touch. Some minor areas may lack depth.	Content is somewhat refined, but lacks consistent depth or personal touch in places.
**Reflection**	Reflection is deep, insightful, and provides a comprehensive understanding of the AI's role in the design process.	Reflection is thoughtful with some insights about AI's role, but might lack depth in places.	Basic reflection on the process with limited insights or depth.

5. Leadership in Implementing AI

**Purpose of the Assignment**	To critically evaluate the considerations and potential challenges of implementing AI in your specific professional context, and to strategize solutions and leadership approaches to address those challenges.<br><br>This assignment is an opportunity for introspection and foresight. While technological advancements like AI can greatly enhance processes and outcomes, thoughtful and strategic leadership is crucial to navigate the associated complexities. Reflect on both the micro and macro aspects of AI implementation and envision yourself as a pivotal leader in this transformative journey.

Due Date: 2/4

Instructions:

1. Contextual Considerations:
   - Begin by briefly describing your professional context
   - Enumerate the specific considerations you, as a leader, would need to keep in mind when introducing AI into this setting. Consider factors such as the organization's mission, stakeholders' needs, existing technological infrastructure, and the nature of services/products offered.
2. Identification of Potential Challenges:
   - Based on your context, identify and elaborate on at least three major challenges you anticipate facing when implementing AI. These challenges can be technical, ethical, logistical, financial, or related to human resources.
3. Addressing Challenges:
   - For each identified challenge:
     a. Propose a strategic solution or approach to mitigate or overcome the challenge.
     b. Describe the leadership skills and strategies you would employ to guide your team or organization through the challenge.
     c. Reflect on any resources, collaborations, or external support you might need to successfully address the challenge.
4. Conclusion:
   - Summarize your findings and reflect on the broader implications of AI implementation in your field. Discuss the balance between staying ahead technologically and ensuring ethical, effective service or product delivery.

Grading Rubric:
Your participation and posts will be evaluated based on:

	Exemplary	Proficient	Basic
**Contextual Insight:** Demonstrated understanding of your specific professional context and its unique considerations.			
**Depth of Challenge Analysis:** Comprehensive identification and elaboration of potential challenges.			
**Solution Strategy:** Practicality, feasibility, and foresight in proposed solutions to address challenges.			
**Leadership Approach**: Clarity and thoughtfulness in described leadership strategies and skills.			
**Overall Cohesion**: Logical flow, clarity, and coherence of the entire document.			

5. Final Reflection

**Purpose of the Assignment**	To introspectively analyze how the course has impacted your understanding and perception of AI, gauge your newfound motivations, and identify the key takeaways that resonated the most with you.<br>This assignment encourages deep introspection and personal engagement with the course content. Consider it an opportunity to consolidate your learning, recognize its influence on your worldview, and chart a path forward in your AI journey.

Due: 2/9

Instructions:

1. Changed Perceptions:
   - Begin your reflection by describing your initial perceptions or beliefs about AI prior to starting this course.
   - Discuss how and in what ways these perceptions have evolved or changed throughout the course.
2. Newfound Motivations:
   - Reflect on how this course has influenced your future endeavors concerning AI. Are there specific areas within AI you are now motivated to explore further, or career paths you're considering?
   - Discuss any immediate actions or steps you plan to take as a result of this motivation.
3. Surprises and Revelations:
   - Were there any topics or pieces of information that took you by surprise or challenged your existing beliefs? What were they and why were they surprising to you?
   - Reflect on why these surprises were significant in your learning journey.
4. Key Takeaways:
   - Identify and elaborate on at least three key takeaways from the course. These could be insights, skills, pieces of knowledge, or overarching themes.
   - Discuss why these takeaways are significant for you and how you envision applying or integrating them into your professional or personal endeavors.
   - Thinking about the way that material was taught during the course or course assignments, what was most impactful to your learning the course content?
   - What methods were least helpful to you?
   - Was there anything unique or different about the ways that the content in this course was delivered? What was that?
   - What impact, if any, did group or peer learning have on your learning about the course content?
   - How do you plan to keep abreast of innovations in AI as you move forward?

Grading Rubric:
Your participation and posts will be evaluated based on:

	Exemplary	Proficient	Basic
**Depth of Reflection:** Demonstrated introspection and depth in your reflections, showing genuine engagement with the course content.			
**Clarity and Cohesion:** Logical flow of thoughts, clear expression of ideas, and overall coherence in your reflections.			
**Solution Strategy:** Practicality, feasibility, and foresight in proposed solutions to address challenges.			
**Specificity:** Providing specific examples, modules, or moments from the course that influenced your perceptions and decisions.			
**Future Implications:** Clear articulation of how the course will influence your future actions, decisions, or career paths concerning AI.			
