# Supplementary material for: Impact of a Practical, Hands-On, Continuing Professional Development Course About AI in Health Care Professions Education on the Perceptions and Behaviors of Health Care Educators: Qualitative Case Study
Source: JMIR Med Educ. 2026 Jun 23;12:e87381. doi: 10.2196/87381 (PMC13290435; doi:10.2196/87381)
Supplement: Multimedia Appendix 2 — Artificial intelligence in health professions education: continuing professional development course presurvey questions. [file mededu-v12-e87381-s002.pdf]

Your Name:

Your answer

What are your personal feelings / emotions associated with the use of artificial intelligence? (Choose all that apply)

- [ ] Excitement
- [ ] Enthusiasm
- [ ] Fear
- [ ] Apprehension
- [ ] Other: ______

What is your prior knowledge of Artificial Intelligence?

- ○ I have no prior knowledge of AI.
- ○ I have basic knowledge about AI (e.g., I understand its basic concepts and have read articles or seen videos about it).
- ○ I have used AI tools or applications in some capacity.
- ○ I have a strong foundation in AI (e.g., I've taken previous courses or have extensive reading on the topic).
- ○ I am proficient in AI (e.g., I have worked on AI projects or have advanced training).

How and what AI technologies have you used?

Your answer

What is your primary motivation for enrolling in this course?

Your answer

What concerns or reservations do you have about the course or the topic?

Your answer

What specific topics or applications of AI in health professions education are you most interested in learning about during this course?

Your answer
