# Supplementary material for: Impact of a Practical, Hands-On, Continuing Professional Development Course About AI in Health Care Professions Education on the Perceptions and Behaviors of Health Care Educators: Qualitative Case Study
Source: JMIR Med Educ. 2026 Jun 23;12:e87381. doi: 10.2196/87381 (PMC13290435; doi:10.2196/87381)
Supplement: Multimedia Appendix 3 — Artificial intelligence in health professions education: continuing professional development course, semistructured interview questions. [file mededu-v12-e87381-s003.pdf]

Interview Questions:

1) Thinking about the way that material was taught during the course or course assignments, what was most impactful to your learning the course content?
2) What methods were least helpful to you?
3) Was there anything unique or different about the ways that the content in this course was delivered? What was that?
4) What impact, if any, did group or peer learning have on your learning about the course content?
5) After the course, how have you used generative AI?
6) What knowledge and skills did you develop during the course that impacted your ability to design, develop, or evaluate the post-course application/project?
7) How do you foresee utilizing AI technologies in your future professional career?
8) What aspects of the course impacted your views on this perspective?
9) Have you investigated any new AI platforms/programs that you have found helpful since the course, would you mind sharing them with future learners?
10) Is there anything related to the research question or topic that you feel that we have not addressed and would like to address now?
