# Supplementary material for: Impact of a Practical, Hands-On, Continuing Professional Development Course About AI in Health Care Professions Education on the Perceptions and Behaviors of Health Care Educators: Qualitative Case Study
Source: JMIR Med Educ. 2026 Jun 23;12:e87381. doi: 10.2196/87381 (PMC13290435; doi:10.2196/87381)
Supplement: Multimedia Appendix 4 — Description of the use of multimodal generative artificial intelligence tools in this paper. [file mededu-v12-e87381-s004.pdf]

The authors acknowledge limited use of multimodal Generative AI tools for two discrete tasks: 1) OpenAI ChatGPT-4o (https://chatgpt.com/, paid version) was used to co-create the sub-themes from the author-identified codes.

A simple prompt asking the tool to generate sub-themes from the author-provided codes. The authors reviewed these sub-themes for accuracy and consistency. These are included in Table 1. The codes and themes were developed by the authors, as described in the manuscript.

Google NotebookLM (https://notebooklm.google/, current Pro version, accessed 2/19/2026) was used to co-create Figure 1 using the manuscript as the source data with author-provided, Google Gemini co-created (https://gemini.google.com, current Pro version, accessed 2/19/2026) meta-prompting, as follows:

Meta-Prompt for Image Generation:

Image Style & Quality: Create a professional, high-quality, clean, isometric infographic suitable for "Figure 1" in an academic medical journal. The background should be a clean, minimalist white or light gray. Photo-realistic images. Not animated or cartoony looking. Images like photographs.

Central Structure: The central element is a large, dynamic, translucent, upward-sweeping spiral helix that *widens* as it ascends from a clean base platform, representing growth. The base of the spiral should be very narrow building as it moves upwards, and widest at the top. Faint lines connect the points of the spiral as it moves upward, representing connectivism networks. The path of the spiral is clean and uncluttered.

Text Labels (Strict Constraint): The *only* text allowed in the entire image are these four specific labels, in a clean sans-serif font. No bullet points, no icon labels, nothing else.

1. **Main Title:** At the very bottom base platform, centered: **AI IN HPE COURSE: EXPERIENTIAL & PEER-BASED LEARNING**
2. **On the bottom section of the spiral:** **MICRO LEVEL: INDIVIDUAL EDUCATOR**
3. **On the middle section of the spiral:** **MESO LEVEL: ORGANIZATION & INSTITUTION**
4. **On the top section of the spiral:** **MACRO LEVEL: GOVERNMENT & SOCIETY**

The Base (Foundation): On a clean, sleek platform at the bottom, depict *only* two distinct groups of healthcare educators (no other people or clutter):

* **Left Side (Experiential Learning):** A group of exactly two healthcare educators in scrubs and lab coats are actively working together on modern laptops at a shared desk. One is looking at and typing on the laptop keyboard the other looks up from the laptop and gestures to the one looking at the laptop.

* **Right Side (Peer-based Learning):** A group of exactly three healthcare educators in professional attire are talking excitedly with each other around a round table, leaning in and gesturing, with one pointing at a tablet on the table.

The Major Call-Out Visuals (High Quality Scenes): Three distinct, detailed, high-quality visual scenes branch off from the spiral layers via clean connecting lines. These scenes must *not* have any text labels:

* **Micro Level Call-out (Right Side):** A single, high-quality scene connected to the "MICRO LEVEL" section, showing one HPE educator in a lab coat using an AI-enabled tool (like a tablet) to precept a trainee in scrubs at a patient's bedside in a hospital room.
* **Meso Level Call-out (Left Side):** A single, high-quality scene connected to the "MESO LEVEL" section, showing one HPE educator standing at a podium, giving a presentation to a large auditorium filled with diverse healthcare professionals listening intently. She should be facing them.
* **Macro Level Call-out (Top Right - Significant Distance):** This image is distinctly separated and placed in the upper right corner, connected to the "MACRO LEVEL" section by a longer line. It is a single, high-quality scene of one HPE educator in formal business attire (suit) presenting a document (a bill or policy) at a podium in a grand legislative capitol building chamber.

Each educator in the micro, meso, macro images should be different ethnicity and gender.
