# Supplementary material for: Impact of a Practical, Hands-On, Continuing Professional Development Course About AI in Health Care Professions Education on the Perceptions and Behaviors of Health Care Educators: Qualitative Case Study
Source: JMIR Med Educ. 2026 Jun 23;12:e87381. doi: 10.2196/87381 (PMC13290435; doi:10.2196/87381)
Supplement: Multimedia Appendix 5 — Additional thematic quotes. [file mededu-v12-e87381-s005.pdf]

Supplemental Document S1. Additional thematic quotes.

Themes	Quotes
AI misconceptions breed fear	Overcome my luddite tendencies that will keep me fearful and cowering in a corner and not using these technologies. P6 (I)<br><br>It makes me feel a bit on the older side and like I am late to the game. I had previously been the one teaching my parents how to use cellphones, and now I feel like a bit of a dinosaur. P11 (A)<br><br>I was worried that trainees would use it to complete assignments, personal statements, etc., essentially making these “useless” in evaluation. P1 (A)<br><br>My feelings of fear and inadequacy related to AI. P8 (A)<br><br>I was still very uncomfortable with using it due to not really knowing how it works and any consequences it may have. P11 (A)<br><br>AI might stunt the learning of students who rely on AI for an easy way to complete assignments. P18 (A)
Education leads to a shift in perspective	I felt as though the curtain had been opened, like in the Wizard of Oz when you realize it was just a man behind the curtain. P8 (A)<br><br>I was extremely naive about the scope of AI and how present it was in my daily life prior to taking this course. P1 (A)<br><br>My perceptions regarding the use of AI in medical education and leadership have continued to evolve as the course has progressed. P1 (A)<br><br>I now realize that most of the field feels the way I do and while there are some out there who have advanced using AI most of us are just figuring out what it is and where it can fit. P8 (A)

	We should not shy away from AI because there are some risks before we look at the potential benefits of its utilization. P10 (A)<br><br>It's exciting to look towards the future to see how I can incorporate, Whether it's teaching Biology again or anything like clinical lab science. P2 (I)<br><br>I was intrigued by some of its features and found it very entertaining. I was amazed at how conversational it was. P10 (A)<br><br>Using it as a shell and then seeing and kind of laughing also where they pull some of the data. Especially if it's very specific for my unit…I wish I would have known where to find that on my own portal. P4 (I)<br><br>My sister is actually a second grade teacher. So I was telling her about it because she didn't know anything about it…She's like, “How do I start story about an inclusive family or whatever her story was.” I was like, “Hell if I know. I don't know anything about that.” So I was like, “Let's try Chat GPT?” P2 (I)
As perspectives shift, learners reach out to their social circles	This process truly felt like a collaborative process that was synergistic and produced something truly awesome. I am actually planning on printing this image in high quality and hanging it somewhere in my office to promote discussion! P15 (A)<br><br>I think it helped me as a parent also because I was reading some of their essays and I was like, “This is not you talking.” Like, “Let's fix this. How do you know about this?” Because they know I’m not very tech savvy. I took a course about this. I know all about it now, so that was fun. P2 (I)<br><br>I’ve been in pretty active conversations with my brother, my brother is an iOS programmer and has done a fair bit just avocationally. So some vocational but even more

	avocationally. Particularly with some of the image generation AI engines and that's where I really want to go play more. P6 (I)<br><br>I did enjoy when we were together as a group as it provided an opportunity to hear about other's experiences implementing AI and their plans moving forward. P1 (A)<br><br>One gentleman had like a totally different idea of how to create his AI model. And I was like mind blown, I would never have thought of that because I'm new to this and so it was nice to be in a group setting and bounce ideas off each other. P2 (I)<br><br>I have taken this a lot into my teaching. I almost always now have a resident or student pulling up an AI tool while we're discussing stuff and using it. P8 (I)<br><br>I have started to follow many social media accounts across many genres and have seen the various utilizations of AI. P10 (A)<br><br>I am hoping to apply this simply by incorporating AI a lot more in various activities I do and trying my best to stay ahead of the rapidly evolving technology. P7 (A)<br><br>One specific project I am now eager to refine is the custom GPT that I developed as part of the course. This GPT helps educators create interactive, case-based PowerPoint slide decks—a tool that could revolutionize how we design and deliver educational content. I have already reached out to colleagues with expertise in coding and AI integration to help troubleshoot some technical challenges I've encountered. P13 (A)
Confidence in AI technologies leads to implementation and advocacy	It'll make your job, maybe a little bit easier in terms of creating your course, content like your syllabus and then how your modules for teaching every week. P2 (I)

	This can help out or reduce some of those times where you're struggling to kind of put things on paper. P4 (I)<br><br>It is easy to have “writer’s block” and it is a really easy way to get thinking a little more creatively and do have another point of view while being very efficient in your own time. P18 (A)<br><br>It just gets you started…I tell this to the residents all the time, when we write, the first person always has the hardest job,…But just like getting that first draft…P8 (A)<br><br>I think I can use AI to manage some of the cognitive load I have in my administrative role. P14 (A)<br><br>In the process of creating an educational course…regarding laboratory data. And I used a lot of what I used in class…I did use like a syllabus outline, 12 week class, and how we break it down for scoring and testing. What module we’re going to do each week. P2 (I)<br><br>What I've been able to do is, I've been able to put the students’ comments that they get from their attendings and ask the tool to match the comments to an EPA for me. And that really lets me bring the EPAs forward in the narrative assessment. “The student is very strong at gathering a history and physical.” At a director retreat. We actually trained all of our site directors to start doing that. Then we have started to try to use a new tool that grades H&Ps. We’re trying to train the tool using a rubric, because there’s a lot of variability across our site directors for grading H&Ps. We’re trying to see if this AI tool can make that more uniform across the board. P8 (I)<br><br>We’ve turned to NeoMind…to look at how others in our field are starting to leverage AI to enhance the health of neonates across the world. P13 (A)<br><br>To avoid being left behind given the very rapid expansion and diversity of AI technology, it is imperative that professional societies, health care delivery systems, leaders

	in medical education, and experts in AI work together to establish best practices for the use of AI in clinical care, education, and leadership. P1 (A)<br><br>We had our Health Professions Education day and we actually focused that…on AI and VR. We had speakers, who came and presented on the use of AI, particularly in medical education because it was Education Day. P1 (A)<br><br>We're putting together a workshop…really trying to pay it forward, so to speak. P6 (A)<br><br>Another key takeaway is the awareness that AI is still underutilized in many areas, primarily due to fears about its ethical and logistical implications. I now see myself as being in a unique position to help combat these fears, particularly in my role as an educator and voice in the field of medical ethics. P13 (A)<br><br>we as leaders have a responsibility to build teams who have the necessary skill sets through recruitment and talent management with professional development opportunities, clearly define a vision of what success looks like, create a culture that values innovation and a growth mindset, while keeping communication open to explore and define (and redefine) ethical boundaries for responsible use of AI technology as it continues to evolve… P12 (A)
